# Variability of Volatile Compounds in the Medicinal Plant *Dendrobium officinale* from Different Regions

**DOI:** 10.3390/molecules25215046

**Published:** 2020-10-30

**Authors:** Jing Hu, Wenxue Huang, Fantao Zhang, Xiangdong Luo, Yaling Chen, Jiankun Xie

**Affiliations:** 1 Laboratory of Plant Genetic Improvement and Biotechnology, Jiangxi Normal University, Nanchang 330000, Jiangxi, China; hujing8963@163.com (J.H.); 15879074628@163.com (W.H.); zhang84004@163.com (F.Z.); xdluolf@163.com (X.L.); 2 Institute of Biophysics, College of Life Sciences, Zhejiang University, Hangzhou 310000, Zhejiang, China

**Keywords:** *Dendrobium officinale*, volatile components, different regions, GC-MS

## Abstract

*Dendrobium officinale* Kimura et Migo, a rare and traditional medicinal plant, contains many nutrients such as polysaccharides, alkaloids, amino acids and so on. Different growth environment and intraspecific hybridization of different germplasm resources lead to large differences in the yield, quality and medicinal value of *D. officinale*. Here, the volatile compounds of *D. officinale* from four producing regions (Zhejiang, Fujian, Yunnan and Jiangxi) were analyzed to provide a certain reference value for the selection of a specific medicinal component in *D. officinale* breeding. Fresh stems of *D. officinale* germplasm resources were collected, and the chemical constituents were determined by gas chromatography-mass spectrometry. A total of 101 volatile compounds were identified, of which esters and alcohols accounted for 23 and 22. Hexacosane is the highest relative content of all volatile components. The highest content of hexacosane was observed in YA1 from Yunnan was 34.41%, and the lowest (23.41%) in JA1 from Jiangxi. Moreover, 5-10 unique substances were determined in different regions. A total of 17 medicinal components were detected, and three unique medicinal components were detected only in YA1, revealing that YA1 can provide raw materials for the application of specific medicinal substances extraction. A total of four toxic components were detected, but no toxic components were detected in JA1 from Jiangxi, suggested that the germplasm resources from Jiangxi could be exploited efficiently for breeding superior *D. officinale* specimens. The results provide a theoretical basis for the collection, protection and utilization of *D. officinale* germplasm resources in different regions.

## 1. Introduction

*Dendrobium* is a perennial herb in the family *Orchidacea* (*Dendrobium Sw.*), widely distributed in Australasia, Oceania and other tropical and subtropical areas. In China, there are 74 *Dendrobium* species and two varieties, and nearly 50 of these species are used in medicine, among which *Dendrobium officinale* Kimura et Migo is commercially valuable. *Dendrobium officinale* Kimura et Migo is a rare and traditional medicinal plant in China, containing many nutrients such as polysaccharides, alkaloids, amino acids, stilbenes, flavonoids, trace elements and so on [1]. Polysaccharides of *D. officinale* exhibit anti-aging, immune-enhancing, anti-inflammatory, liver-protective and anti-tumor activities [2]. The biological activities of alkaloid have been proved anti-oxidation, treat cell damage, anti-inflammation, liver-protection, anti-neurodegeneration and so on [3,4]. The medicinal value of *D. officinale* is recognized by the public and market demand has been steadily on the increase, but wild *D. officinale* usually grows on nutrient-poor cliffs or tree trunks on mountains close to one kilometer above sea level and prefers to grow on wet rocks, indicating that it has very strict requirements for its habitats, and mainly distributed in Southwest Anhui, Eastern Zhejiang, Jiangxi, Western Fujian, Northwest Guangxi, Sichuan and Southeast Yunnan [5]. Meanwhile, over-exploitation led to the depletion of its wild resources and is now listed as an IUCN critically endangered plant [6,7].

In the long-term evolution process, the self-incompatibility mechanism of reproductive isolation was formed, which showed that the pistils and stamens develop normally and mature at the same time and the seeds can’t be produced after self-pollination or cross pollination with the same genotype [8]. The fine varieties of *D. officinale* can only be preserved by intraspecific hybridization or asexual propagation. At present, most of the *D. officinale* in the market are generated through intraspecific hybridization of different categories, results in different quality and medicinal value of *D. officinale* in different regions [9]. In addition, different distribution regions and growth environment may lead to a large gap in the yield and quality of *D. officinale* [10]. Therefore, it is of great significance to find, collect and cultivate of *D. officinale* germplasm resources with different qualities for its industrialization.

Here, we collected fresh stem of *D. officinale* germplasm resources from different regions (Zhejiang, Fujian, Yunnan and Jiangxi), the chemical constituents of *D. officinale* from different regions were determined by a gas chromatography-mass spectrometry (GC-MS)-based metabolomic approach. The medicinal components and toxic substances of *D. officinale* from the four producing regions were analyzed to provide a certain reference value for the selection of a specific medicinal component in *D. officinale* breeding. The results should provide theoretical basis for the collection, protection and utilization of *D. officinale* germplasm resources in different regions.

## 2. Results

### 2.1. Metabolic Profiling of D. officinale in Different Regions

**Figure 1:**
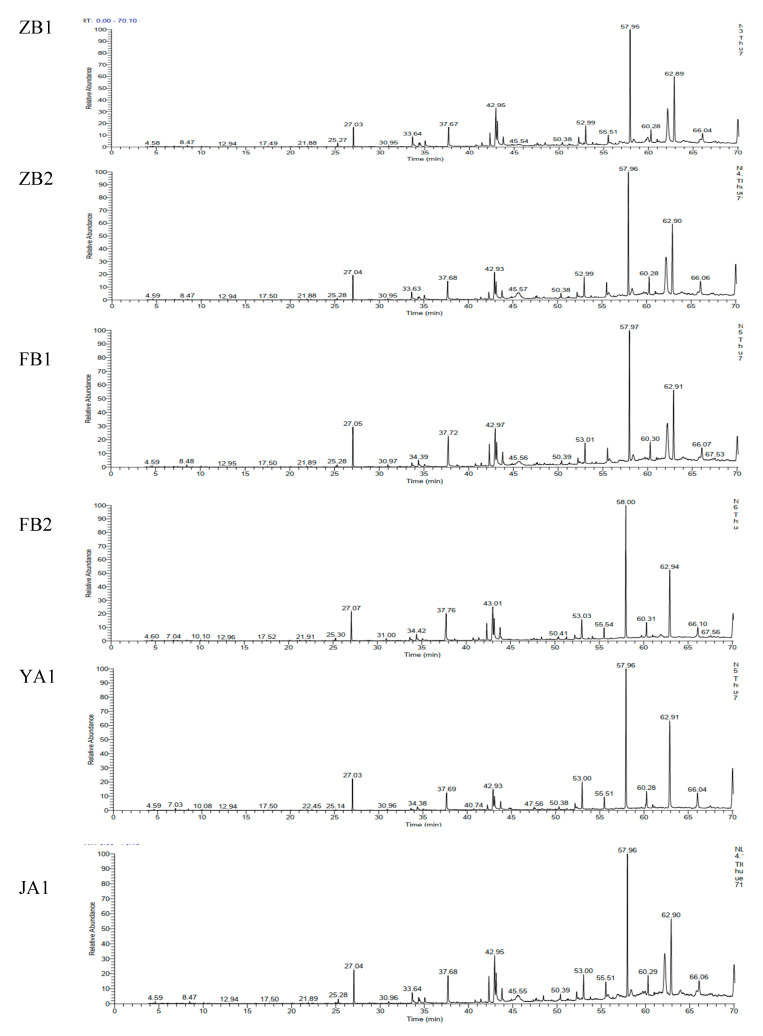
The total ion chromatograms of volatile compounds determined by GC-MS in the *D. officinale* of ZB1, ZB2, FB1, FB2, YA1 and JA1.

To investigate whether the chemical composition of *D. officinale* is associated with its origin, we analyzed six samples of *D. officinale* from different territory, ZB1 (purple stems, Zhejiang), ZB2 (green stems, Zhejiang), FB1 (purple stems, Fujian), FB2 (green stems, Fujian), YA1 (purple stems, Yunnan) and JA1 (purple stems, Jiangxi). We analyzed these stems using a GC-MS-based metabolomics approach, and the total ion chromatographic (TIC) diagram is shown in Figure 1. The TIC for ZB1 and ZB2 are basically the same, and FB1 was similar to FB2. There was a significant difference in the intensity of compound peaks in the TIC for the samples from different regions, especially the peaks with retention time between 45–70 min (Figure 1).

Among the 101 identified metabolites from the six samples, three were identified as aldehydes, 22 were alcohols, 23 were esters, one was a terpene, four were ketones, four were phenols, nine were organic acids, six were olefins, 17 were alkanes and their derivatives and 12 were other compounds (Table 1). In ZB1 and ZB2, the most volatile compounds were alcohols, and there were 11 volatile compounds in both groups (Table 1). The varieties of other constituent categories except alcohols were also similar. In FB1 and FB2, esters were the most volatile compounds, and the kinds of other composition categories were similar.

Among the volatile components of YA1, esters were the highest, but in JA1, alcohols were the highest. The results showed that there was no significant difference in the composition categories of volatile components in the stems of *D. officinale* with different colors in the same region, but there were significant differences in the composition categories of volatile components in different regions, such as esters.

### 2.2. Analysis of Relative Content of Volatile Components in D. officinale

The volatile components and their relative contents in the stem of *D. officinale* are listed in the Supplementary Materials (Table S1). The substances with the largest proportion of volatile components were alkanes and their derivatives, which were 52.64%, 56.74%, 49.86%, 58.17%, 50.32% and 72.40% respectively in ZB1, ZB2, FB1, FB2, YA1 and JA2 (Table S1). Hexacosane shows the highest relative content of all volatile component, the highest content being 34.41% in YA1 and the lowest 23.41% in JA1 (Table S1). In addition to hexacosane, there were five kinds of volatile substances with a relative content of more than 2%, which were triacontane (highest level 27.42% in YA1, and the lowest 17.29 in FB1), tetratriacontane (highest 8.42% in YA1, and lowest 2.55% in FB2), phosphoric acid, dibutyl 1,1-dimethyl-2,2,3,3-tetrafluoro propyl ester (the highest level was 5.02% in YA1, and the lowest was 2.87% in ZB1), 9-octadecynoic acid (the highest content 7.32% in ZB1, and the lowest was 4.06% in YA1), palmitic acid (the highest in FB1 (6.09%), and the lowest in ZB1 (3.21)). Interestingly, *D. officinale* from different regions has 5–10 unique substances, accounting for no more than 5.5% of the total, that revealed that the categories and relative contents of volatile components from different regions vary greatly.

### 2.3. Medicinal Components of D. officinale from Different Regions

**Figure 2:**
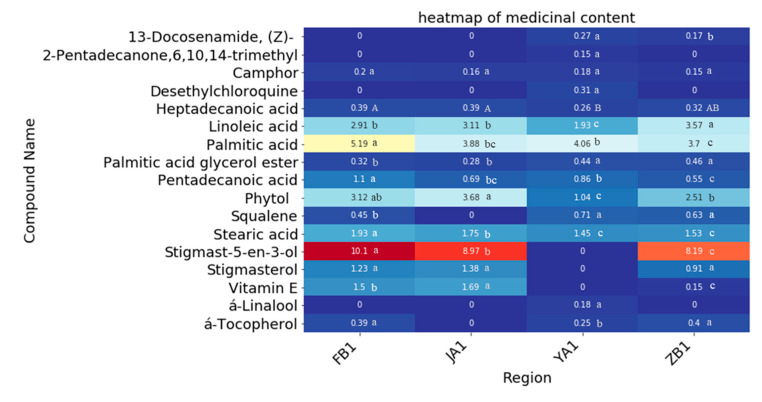
The relative levels of medicinal components differed in *D. officinale* in four different regions from GC-MS data (ZB1, FB1, YA1 and JA1). Capital letters A and B indicate significant differences at *p* < 0.01, and lowercase letters a, b, and c indicate extremely significant differences at *p* < 0.05.

Xiang [11] found that *D. officinale* contains many medicinal components, such as vitamin E, phytol, hexadecanoic acid and other bioactive components. As shown in Figure 2, a total of 17 medicinal components were detected in the volatile substances, including α-linalool, camphor, pentadecanoic acid, 6,10,14-trimethyl-2-pentadecanone, palmitic acid, heptadecanoic acid, phytol, linoleic acid, stearic acid, desethylchloroquine, vitamin E, stigmasterol, stigmast-5-en-3-ol, palmitic acid glycerol ester, (*Z*)-13-docosenamide, squalene and α-tocopherol. The relative content of stigmast-5-en-3-ol was the highest among these 17 medicinal components. Moreover, the highest content of stigmast-5-en-3-ol in FB1 was 10.05%, which was significantly higher than that in ZB1, FB1 and JA1 (*p* < 0.01). Unexpectedly, stigmast-5-en-3-ol was not detected in YA1 from Yunnan. The contents of palmitic acid in YA1 were significantly higher than that in the other three regions, but the content of phytol and linoleic acid in YA1 was the lowest. The content of vitamin E in the Jiangxi sample was higher than that in ZB1 and FB1, and it was not detected in YA1. (*Z*)-13-Docosenamide was found in the ZB1 and YA1 samples, and α-linalool, 6,10,14-trimethyl-2-pentadecanone and desethylchloroquine were detected only in YA1.

### 2.4. Toxic Components of D. officinale from Different Regions

**Figure 3:**
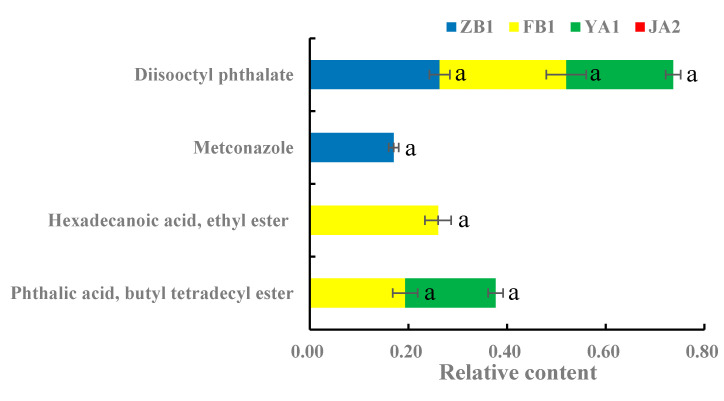
The relative content of toxic components in *D. officinale* in four different regions from GC-MS data (ZB1, FB1, YA1 and JA1). The letters a and b indicate significant difference at *p* < 0.05. Since the sample data is 0, b was not indicated in the figure.

As shown in Figure 3, the categories and relative contents of toxic rational components of *D. officinale* in different regions were different. A total of two components in ZB1 are considered to be toxic, with a total relative content of 0.43%. The relative content of diisooctyl phthalate was the highest (0.26%) and that of metconazole was the lowest (0.17%), which was significantly higher than that of FB1, JA1 and YA1 (*p* < 0.05). A total of three toxic components were detected in FB1, with a total relative content of 0.71%, which was the highest among the four groups. Among them, the relative contents of diisooctyl phthalate and hexadecanoic acid ethyl ester were the highest, both 0.26%. The relative content of phthalic acid butyl tetradecyl ester with the lowest relative content was only 0.19%, which was significantly higher than that of ZB1 and JA1 (*p* < 0.05). A total of two components in YA1 were considered to be toxic, with a total relative content of 0.40%. The relative content of diisooctyl phthalate was the highest (0.22%) and that of phthalic acid butyl tetradecyl ester was the lowest, which was not significantly different from that of FB1, but significantly higher than that of ZB1 and JA1 (*p* < 0.05). Interestingly, no toxic components were detected in JA1.

## 3. Discussion

Previous studies have shown that the growth and chemical components of *D. officinale* are significantly different under different growth conditions, light, growth years and harvest ways [12,13,14]. Bai [15] reported that there were remarkable differences in morphology including stem height and diameter, amount of lateral bud, and texture of stem etc. from five regions, and the content of polysaccharides was also significantly different. However, few studies have focused on the chemical composition differences of *D. officinale* from different regions. In this study, the categories and contents of volatile compounds in *D. officinale* from different regions were different, with the samples from Zhejiang Province (ZB1 and ZB2) containing the most, and those from Fujian Province (FB1 and FB2) the least (Table 1). Cheng [16] found that short light/dark cycle can increase the biomass and polysaccharide yield of *D. officinale*. Different growth condition and symbiotic bacteria in the roots of *D. officinale* may also lead to differences in volatile components [17], so the categories and contents of volatile compounds in *D. officinale* from different regions may be caused by the different temperature, humidity, soil conditions or sunshine duration in the different regions.

The volatile components of plants mainly contain terpenoids, which have strong bactericidal, anti-inflammatory, analgesic and anticancer effects and are an important component of medicinal plants [18]. A total of 17 medicinal components were detected in the volatile substances from the four different regions, and the content of stigmast-5-en-3-ol was the highest except in sample YA1 from Yunnan, where it was not detected (Figure 2). Stigmast-5-en-3-ol belongs to the phytosterols. Phytosterols, triterpene compounds with a cyclopentane polyhydrophenanthrene as the main structure, are an important part of plant cell biofilm and widely exist in all kinds of plants [19]. The molecular structure of phytosterols is similar to that of cholesterol, which can inhibit the intestinal absorption of cholesterol, thereby reducing the level of cholesterol in the blood and reducing the risk of cardiovascular disease [20]. Moreover, the content of organic acids in FB1 from Fujian Province was highest (Figure 2.). Linoleic acid is an essential fatty acid for human body and has obvious anticancer effects under the joint action of other compounds. It can also reduce cholesterol and triglycerides in the blood, reduce blood viscosity, enhance the body’s defense system functions, etc. [21]. Palmitic acid and stearic acid can reduce the content of cholesterol in serum. Therefore, it has been suggested that replacing dietary lauric acid and myristic acid with palmitic acid and oleic acid may be beneficial for the treatment of thrombosis [22]. In addition, some studies have shown that pentadecanoic acid can improve hyperinsulinemia, protect islet cells and improve the inflammatory state of diabetic mice [23]. Heptadecanoic acid is an effective drug against NSCLC cells in vitro [24].

Interestingly, many unique medicinal ingredients including α-linalool, 6,10,14-trimethyl-2-pentadecanone and desethylchloroquine were detected only in sample YA1 from Yunnan Province (Figure 2.). α-Linalool is a natural flavor and fragrance that exists in many medicinal plants. It has medical and health care functions and can be used against dental caries, for deworming and an insecticide [25]. 6,10,14-Trimethyl-2-pentadecanone is an intermediate in the production of vitamin E acetate. The results provide a theoretical basis for the development of medicinal value of *D. officinale* in different regions and the utilization of *D. officinale* resources with specific efficacy.

At present, most of the *D. officinale* on the market is artificially cultivated, and thus may contain pesticide residues or other toxic ingredients. A total of four toxic components were detected, including phthalic acid butyl tetradecyl ester, hexadecanoic acid ethyl ester, metconazole and diisooctyl phthalate. There were three categories of toxic components in FB1 from Fujian, followed by ZB1 from Zhejiang and YA1 from Yunnan (Figure 3). Diisooctyl phthalate was detected in samples from all regions except in JA1 from Jiangxi. Diisooctyl phthalate, identified as the fourth category of toxic chemicals, is a kind of plasticizer and widely used in plastic products, building materials, food packaging, cosmetics and household materials [26]. Human intake of high doses of plasticizers not only harms the reproductive system, but also causes cardiovascular disease and may even cause cancer [27]. Metconazole is a fungicide that interferes with the synthesis of fungal cell membranes [28]. As mentioned, unexpectedly, no toxic components were detected in JA1 from Jiangxi. In addition, the categories of volatile compounds and the content of medicinal components in JA1 from Jiangxi were also high. Xie [29] showed that the origin of market-collected individuals was derived from Zhejiang and the germplasm resources from Jiangxi were well preserved, so the germplasm resources from Jiangxi should be exploited efficiently for breeding superior *D. officinale* individuals.

## 4. Material and Methods

### 4.1. Plant Materials

Our previous results showed that genetic diversity of *D. officinale* varies between different regions, most of the *D. officinale* in the market were derived from Zhejiang and the germplasm resources from Jiangxi were well preserved [29], so *D. officinale* germplasm resources from Zhejiang, Yunnan, Jiangxi and Fujian were collected as experimental materials. All the experimental materials were cultivated in the greenhouse. The seedlings were transplanted into pots and cultivated in the growth chamber with a light: dark cycle of 12 h each at 15 to 28 °C and a relative humidity of 70 to 80%. Pine bark was used as the substrate. Biennial materials were collected in February 2018.

Zhu [30] found that the stems of *D. officinale* can have two colors (purple and green) and different morphology. In addition, the morphological characteristics of *D. officinale* are correlated with the contents of polysaccharides and total alkaloids. In order to explore whether the color of the stem is related to the content of volatile substances, *D. officinale* samples from the same area were divided into two categories according to the color of the stem. *D. officinale* with purple stem from Zhejiang was named ZB1. Correspondingly, *D. officinale* with green stem from Zhejiang was named ZB2. Similarly, *D. officinale* with purple stem from Fujian was named FB1, while *D. officinale* with green stem from Fujian was named FB2. Since no *D. officinale* with green stem was found in Yunnan and Jiangxi, the two groups were named YA1 and JA1, respectively.

### 4.2. Sample Preparation for GC–MS Analysis

The samples with uniform thickness and no insect eye and mildew were selected, defoliated and cut off. The samples of fresh stem of *D. officinale* were dried in an oven at 50 °C for 15 days. The dried stems were ground to powder. One hundred g of powder from each group was placed in a round bottom flask with 500 mL of *n*-hexane (analytical purity) and stirred evenly. After extracting twice by the condensation reflux method, the liquid supernatant was obtained by decompression filtration. The *n*-hexane was removed by rotary evaporation and the paste can be used as a backup. Each extraction was repeated three times.

### 4.3. GC–MS Analysis

Volatile compounds of *D. officinale* were extracted with an *n*-hexane reflux method [31], then transferred into a glass bottle for GC-MS analysis (Trace1300/ISQ, Thermo Fisher Scientific, Waltham, MA, USA). Each group (ZB1, FB1, YA1, and JA1) contains three biological replicate times. On microliter of each sample was injected into the GC-MS at 250 °C in a shunt mode (20:1) with carrier gas (>99.999% helium) at a flow rate of 1 mL/min, and separated by a HP-5 MS (30 m × 0.25 mm, 0.25 μm) capillary column. The temperature was held isothermally for 3 min at 80 °C, then raised to 260 °C at a rate of 3 °C per minute, and held for 10 min. The transmission line temperature was set to 280 °C, and the ion source temperature was set to 280 °C. The mass range analyzed was from *m/z* 50 to 650 [32].

### 4.4. Data Processing and Multivariate Statistical Analysis

The obtained GC-MS data was processed by the Xcalibur 2.2 SP1 (Thermo Fisher Scientific, Waltham, MA, USA). The compounds in *D. officinale* stems were identified by matching the MS mass spectrogram with the mass spectrogram in the NIST library and referring to the retention time and molecular formula in the relevant literature. Then, using the Xcalibur chromatography workstation data processing system, the relative content of each compound was calculated according to the peak regions normalization method. SPSS23.0 (International Business Machines Corporation, Armonk, NY, USA) was used for one-way ANOVA, and Tukey B and the least significant difference method (LSD) were used for significance testing to analyze the significant differences (*p* value of <0.01) of drugs or toxic components among the four groups.

## 5. Conclusions

In this study, the categories and contents of volatile compounds in *D. officinale* from different regions are variety, and the categories from Zhejiang Province (ZB1 and ZB2) are the most, and those from Fujian Province (FB1 and FB2) are the least. There are unique substances in *D. officinale* from different regions, showing that we can selectively cultivate *D. officinale* according to the different unique substances desired A total of 17 medicinal components were detected. The highest content of stigmast-5-en-3-ol (10.05%) was observed in FB1 from Fujian, which was significantly higher than that in ZB1, FB1 and JA1 (*p* < 0.01), revealing that FB1 can provide high quality raw materials for applications using stigmast-5-en-3-ol. Interestingly, no toxic components were detected in JA1 from Jiangxi, suggested that the germplasm resources from Jiangxi should be exploited efficiently for breeding superior *D. officinale* individuals.

## Figures and Tables

**Table 1:** Categories and relative contents of volatile components in *Dendrobium officinale* from different regions.

Place of Origin	Sample Number	Relative Content % (the Numbers of Volatile Components)									
		Aldehyde	Alcohols	Esters	Terpenes	Ketones	Phenols	Organic Acids	Olefins	Alkanes and Their Derivatives	Other
Zhejiang	ZB1	0.41(1a)	14.25(11ab)	10.00(8c)	0.62(1a)	0.51(3a)	0.54(2a)	17.28(8a)	0.88(4a)	52.64(10a)	3.11(4ab)
	ZB2	0.83(1a)	16.57(11ab)	8.53(7c)	0.55(1a)	0.28(1a)	2.83(1a)	12.32(8a)	0.48(2ab)	56.74(10a)	0.86(4ab)
Fujian	FB1	0.46(1a)	17.92(12a)	9.59(14a)	0.43(1a)	0.50(2a)	1.89(2a)	18.26(8a)	0.28(1b)	49.86(8ab)	0.46(2b)
	FB2	0.91(1a)	5.18(6d)	10.57(16a)	0.46(1a)	0.57(1a)	0.63(3a)	21.49(8a)	0.55(3ab)	58.17(7b)	1.50(5a)
Yunnan	YA1	0.82(1a)	2.44(7cd)	8.39(11b)	0.66(1a)	0.54(3a)	0.39(2a)	11.25(8a)	0.51(1b)	72.40(9ab)	1.30(5a)
Jiangxi	JA1	0.60(2a)	16.74(9bc)	10.40(8c)	0(0a)	0.41(2a)	1.64(1a)	17.1(8a)	1.06(3ab)	50.32(9ab)	0.75(2b)

Note: Different letters indicate significant differences at *p* < 0.05.

## Supplementary Materials

The following are available online, Table S1: Categories and relative contents of volatile components in Dendrobium officinale from different regions.
