# Supplementary material for: Variability of Volatile Compounds in the Medicinal Plant Dendrobium officinale from Different Regions
Source: Molecules. 2020 Oct 30;25(21):5046. doi: 10.3390/molecules25215046 (PMC7663752; doi:10.3390/molecules25215046)
Supplement: Supplementary file 1 [file molecules-25-05046-s001.pdf]

Supplement Table S1. Categories and relative contents of volatile components in *Dendrobium officinale* from different regions

No.	Compound Name	Molecular Formula	Relative Content%					
			Zhejiang		Fujian		Yunnan	Jiangxi
			ZB1	ZB2	FB1	FB2	YA1	JA2
Aldehyde								
1	Hexadecanal	$C_{16}H_{32}O$	-	-	-	0.2	-	-
2	Octadecanal	$C_{18}H_{36}O$	0.41	0.83	0.46	0.71	0.82	0.38
Alcohols								
3	á-Linalool	$C_{10}H_{18}O$	-	-	-	-	0.24	-
4	E-2-Tetradecen-1-ol	$C_{14}H_{28}O$	-	-	0.17	-	-	-
5	1-Pentadecanal	$C_{15}H_{30}O$	-	-	-	-	-	0.22
6	Hexadecen-1-ol, trans-9-	$C_{16}H_{32}O$	0.26	0.18	-	-	-	-
7	1-Hexadecanol	$C_{16}H_{34}O$	0.63	0.39	-	0.57	0.13	0.48
8	7-Methyl-Z-tetradecen-1-ol	$C_{17}H_{32}O_2$	-	-	-	0.43	-	0.32
9	18-Nonadecen-1-ol	$C_{19}H_{38}O$	-	0.21	-	-	-	-
10	n-Heptadecanol-1	$C_{19}H_{40}O$	-	-	0.46	-	-	-
11	Phytol	$C_{20}H_{40}O$	2.26	1.17	3.05	3.05	0.95	3.23
12	E,E,Z-1,3,12-Nonadecatriene-5,14-diol	$C_{19}H_{34}O_2$	-	-	0.55	-	-	-
13	2-cis-9-Octadecenyloxyethanol	$C_{20}H_{40}O_2$	0.28	-	-	-	0.14	-
14	2-Octadecyloxyethanol	$C_{20}H_{42}O_2$	0.26	0.31	0.22	-	-	-
15	n-Tetracosanol-1	$C_{24}H_{50}O$	-	-	-	0.25	-	-
16	4,6-Cholestadien-3á-ol	$C_{27}H_{44}O$	0.38	-	0.47	0.38	0.37	0.63
17	1-Heptacosanol	$C_{27}H_{56}O$	-	-	-	-	0.49	-
18	5-Cholestene-3-ol, 24-methyl-	$C_{28}H_{48}O$	-	0.38	-	-	-	-
19	5à-Cholestan-3á-ol, 2-methylene	$C_{28}H_{48}O$	-	-	0.35	-	-	-
20	ç-Ergostenol	$C_{28}H_{48}O$	-	-	-	-	-	0.37
21	Ergost-5-en-3á-ol	$C_{28}H_{48}O$	0.36	0.72	0.7	-	-	0.53
22	Stigmasterol	$C_{29}H_{48}O$	0.89	1.4	1.28	-	-	1.36
23	Stigmast-7-en-3-ol, (3á,5à,24S)-	$C_{29}H_{50}O$	0.55	0.4	0.53	-	-	0.54

24	Stigmast-5-en-3-ol	$C_{29}H_{50}O$	8.2	11.26	9.96	0.75	-	8.97
25	1-Heptatriacotanol	$C_{37}H_{76}O$	-	-	0.18	0.18	0.12	0.63
Eters								
26	Phosphoric acid, dibutyl 1,1-dimethyl-2,2,3,3-tetrafluoro propyl ester	$C_{13}H_{25}F_4O_4P$	2.87	3.4	4.94	4.97	5.02	3.85
27	4-Methoxybenzyl phenyl carbonate	$C_{15}H_{14}O_4$	0.57	-	0.32	0.58	0.28	0.75
28	Z-(13,14-Epoxy)tetradec-11-en-1-ol acetate	$C_{16}H_{28}O_3$	-	-	0.39	-	-	-
29	Ethyl 13-methyl-tetradecanoate	$C_{17}H_{34}O_2$	-	-	-	0.14	-	-
30	Hexadecanoic acid, ethyl ester	$C_{18}H_{36}O_2$	-	-	0.23	0.29	-	-
31	Palmitic acid glycerol ester	$C_{19}H_{38}O_4$	0.46	0.33	0.31	0.29	0.44	0.28
32	Linolein, 2-mono	$C_{21}H_{38}O_4$	-	0.89	-	-	-	-
33	9,12-Octadecadienoic acid(Z,Z)-, trimethylsilyl ester	$C_{21}H_{40}O_2Si$	0.35	-	-	-	-	-
34	n-Propyl 9,12-octadecadienoate	$C_{21}H_{38}O_2$	1.38	-	0.89	0.8	1.11	1.16
35	9,12,15-Octadecatrienoic acid,2,3-dihydroxypropyl ester,(Z,Z,Z)-	$C_{21}H_{36}O_4$	-	-	-	0.15	-	-
36	Phytol, acetate	$C_{22}H_{42}O_2$	3.68	2.67	0.99	1.08	0.58	2.79
37	1-Heneicosyl formate	$C_{22}H_{44}O_2$	-	-	0.21	0.25	-	0.23
38	Methyl 19-methyl-eicosanoate	$C_{22}H_{44}O_2$	-	0.18	0.23	0.31	-	-
39	Sulfurous acid,cyclohexylmethyl hexadecylester	$C_{23}H_{46}O_3S$	-	0.29	0.22	-	0.18	-
40	1,3-Benzenedicarboxylic acid,bis(2-ethylhexyl) ester	$C_{24}H_{38}O_4$	-	-	-	0.15	0.12	-
41	Diisooctyl phthalate	$C_{24}H_{38}O_4$	0.24	-	0.25	0.57	0.2	-
42	(Z)-14-Tricosenyl formate	$C_{24}H_{46}O_2$	0.45	0.77	0.27	0.26	-	1.02

43	Docosanoic acid, ethyl ester	$C_{24}H_{48}O_2$	-	-	-	0.13	-	-
44	Phthalic acid, butyl tetradecyl ester	$C_{26}H_{42}O_4$	-	-	0.17	-	0.17	-
45	Oleic acid,3-(octadecyloxy)propyl ester	$C_{39}H_{76}O_3$	-	-	0.17	-	-	-
Terpenes								
46	Squalene	$C_{30}H_{50}$	0.62	0.55	0.43	0.46	0.66	-
Ketones								
47	Camphor	$C_{10}H_{16}O$	0.17	-	0.22	-	0.2	0.19
48	2-Hydroxy-4-isopropyl-7-methoxytropone	$C_{11}H_{14}O_3$	0.62	0.21	0.3	0.47	-	0.55
49	Ethanone,2-(4-methoxyphenyl)-1-(4-methylsulfonylphenyl)-	$C_{16}H_{16}O_4S$	0.16	0.28	0.28	0.57	0.22	0.22
50	2-Pentadecanone,6,10,14-trimethyl	$C_{18}H_{36}O$	-	-	-	-	0.12	-
Organic acids								
51	Myristic acid	$C_{14}H_{28}O_2$	0.22	0.18	0.32	0.43	0.19	0.26
52	Pentadecanoic acid	$C_{15}H_{30}O_2$	0.55	0.37	1.18	1.15	0.79	0.68
53	Palmitic acid	$C_{16}H_{32}O_2$	3.62	3.21	5.12	6.09	4.05	3.78
54	Heptadecanoic acid	$C_{17}H_{34}O_2$	0.32	0.2	0.48	0.51	0.25	0.42
55	(Z,Z,Z)-9,12,15-Octadecatrienoic acid	$C_{18}H_{30}O_2$	0.21	0.17	0.24	0.15	0.47	-
56	9-Octadecynoic acid	$C_{18}H_{32}O_2$	7.32	4.68	5.98	7.22	4.06	6.67
57	Linoleic acid	$C_{18}H_{32}O_2$	3.53	2.18	2.95	3.62	1..92	3.18
58	Stearic acid	$C_{18}H_{36}O_2$	1.51	1.33	1.99	2.32	1.44	1.74
59	Retinoic acid,5,6-epoxy-5,6-dihydro	$C_{20}H_{28}O_3$	-	-	-	-	-	0.37
Olefins								
60	9-Eicosene, (E)-	$C_{20}H_{40}$	-	-	-	0.18	-	-
61	10-Heneicosene (c,t)	$C_{21}H_{42}$	0.26	-	-	-	-	0.19
62	1,21-Docosadiene	$C_{22}H_{42}$	-	-	-	0.16	-	-
63	9-Hexacosene	$C_{26}H_{52}$	0.26	-	-	-	-	-
64	17-Pentatriacontene	$C_{35}H_{70}$	0.16	0.3	0.28	0.21	0.51	0.69

Alkanes and Their Derivatives								
65	1-Bromo-4-bromomethyldecane	$C_{11}H_{22}Br_2$	0.17	-	-	-	-	-
66	Dodecane	$C_{12}H_{26}$	-	-	-	0.14	-	-
67	2-Bromooctadecanal	$C_{18}H_{35}BrO$	0.17	-	-	-	0.16	-
68	Nonadecane	$C_{19}H_{40}$	0.15	0.23	0.22	0.15	-	0.2
69	2-Methylnonadecane	$C_{20}H_{42}$	-	-	-	-	0.14	0.4
70	Tricosane	$C_{23}H_{48}$	0.25	0.4	0.33	0.86	0.35	0.39
71	Tetracosane, 1-bromo-	$C_{24}H_{49}Br$	-	-	-	-	-	0.18
72	Tetracosane	$C_{24}H_{50}$	0.75	0.86	-	-	0.63	0.97
73	Heptadecane, 9-octyl-	$C_{25}H_{52}$	-	-	0.65	-	-	-
74	1-Hexacosene	$C_{26}H_{52}$	0.2	0.18	-	-	-	0.18
75	Hexacosane	$C_{26}H_{54}$	25.36	26.13	24.9	32.07	34.41	23.41
76	11-Butyldocosane	$C_{26}H_{54}$	1.32	-	-	-	-	-
77	Octadecane,3-ethyl-5-(2-ethylbutyl)-	$C_{26}H_{54}$	-	0.59	-	-	-	-
78	Triacontane	$C_{30}H_{62}$	18.01	20.26	17.29	22.02	27.42	19.15
79	Docosane, 11-decyl-	$C_{32}H_{66}$	-	0.57	-	-	-	-
80	Tetracosane, 11-decyl-	$C_{34}H_{70}$	-	0.42	0.44	-	0.58	-
81	Tetratriacontane	$C_{34}H_{70}$	6.3	5.75	5.25	2.55	8.42	4.38
82	1,54-Dibromotetrapentacontane	$C_{54}H_{108}Br_2$	0.16	1.53	0.78	0.38	0.29	1.24
Phenols								
83	Phenol, 2,6-dimethyl-4-nitro-	$C_8H_9NO_3$	-	-	-	0.17	-	-
84	3-n-Butylthiophene-1,1-dioxide	$C_8H_{12}O_2S$	-	-	-	-	0.36	-
85	á-Tocopherol	$C_{28}H_{48}O_2$	0.39	-	0.37	0.33	0.25	-
86	Vitamin E	$C_{29}H_{50}O_2$	0.15	2.83	1.52	-	-	1.64
Other								
87	2(4H)-Benzofuranone,5,6,7,7a-tetrahydro-4,4,7a-trimethyl-, (R)-	$C_{11}H_{16}O_2$	-	-	-	-	0.13	-
88	Benzene,(3-iodo-1-methoxybutyl)-	$C_{11}H_{15}IO$	-	0.29	-	-	-	-
89	Piperazine, 1-ethyl-4-phenyl-	$C_{12}H_{18}N_2$	-	-	-	-	0.21	-

90	Decanamide,N-(2-hydroxyethyl)-	$C_{12}H_{25}NO_2$	-	0.14	-	0.18	-	-
91	2H-Pyran-2-one,tetrahydro-6-nonyl	$C_{14}H_{26}O_2$	-	-	-	0.17	0.16	-
92	Bis-tert.-butylquinone	$C_{14}H_{20}O_2$	-	-	-	0.13	-	-
93	2,4-Bis(tert-butyl)-phenol	$C_{14}H_{22}O$	-	-	-	0.13	0.14	-
94	Desethylchloroquine	$C_{16}H_{22}ClN_3$	-	-	-	-	0.32	-
95	Metconazole	$C_{17}H_{22}ClN_3O$	0.18	0.15	-	-	-	-
96	Oxirane, hexadecyl-	$C_{18}H_{36}O$	-	0.22	-	-	-	-
97	Oxirane, heptadecyl-	$C_{19}H_{38}O$	2.15	-	-	-	-	-
98	Pyrrolidine,1-(1-oxo-7,10-octadecadienyl)-	$C_{22}H_{39}NO$	0.18	-	-	-	-	-
99	13-Docosenamide, (Z)-	$C_{22}H_{43}NO$	0.19	-	-	0.47	0.27	-
100	Icosanoic anhydride	$C_{40}H_{78}O_3$	-	-	0.16	0.25	0.14	0.2
101	Docosanoic anhydride	$C_{44}H_{86}O_3$	0.15	-	-	-	-	-

Note: “-” indicated not checked out.
